# Residual insomnia in major depressive disorder: a systematic review

**DOI:** 10.3389/fpsyt.2023.1190415

**Published:** 2023-06-16

**Authors:** Aleksander Kwaśny, Adam Włodarczyk, Alicja Dywel, Joanna Szarmach, Olivia Strandberg, Wiesław Jerzy Cubała

**Affiliations:** Department of Psychiatry, Faculty of Medicine, Medical University of Gdańsk, Gdańsk, Poland

**Keywords:** residual symptoms, residual insomnia, depression, major depressive disorder, psychopharmacology, psychotherapy, cognitive-behavioral therapy, behavioral activation

## Abstract

**Introduction:**

The ultimate goal in major depressive disorder (MDD) treatment is recovery. A proportion of MDD patients with formal remission experience persistent difficulties, which impair their daily functioning. Residual insomnia is one of the most common residual symptoms. Patients with residual insomnia experience relapse significantly earlier and have a poor prognosis. Little is known about possible ways of treatment and what subtype of insomnia is mostly reported.

**Methods:**

A systematic literature review was carried out in PubMed and Web of Science to synthesize the current status of knowledge about effective treatment methods and insomnia subtypes in residual insomnia in MDD.

**Results:**

A few non-pharmacological treatment methods e.g., Cognitive Behavioral Therapy for Insomnia (CBT-I), Mindfulness-Based Cognitive Therapy (MBCT), behavioral activation (BA) and pharmacological methods (gabapentin, clonazepam) have proven to mitigate residual insomnia. Cognitive Behavioral Therapy for Depression (CBT-D) ameliorates insomnia complaints to a limited extent. Mid-nocturnal insomnia is the most common residual insomnia subtype in MDD patients.

**Conclusion:**

Residual insomnia is a very common complaint and most often appears as mid-nocturnal insomnia. Scarce data points out the benefits from pharmacotherapy, psychotherapy, and BA. More research is needed.

## 1. Introduction

Major depressive disorder (MDD) is a challenging disorder and is one of the leading causes of years lived with disability worldwide (1). In 2015 over 16 million people in the United States reported depressive symptoms (2). Even up to 60% of patients may experience work impairment with an average of over 4 workdays lost or unproductive due to the illness (3). MDD increases overall mortality and is a risk factor for a number of comorbid medical disorders such as stroke, diabetes, or myocardial infarction (4–8). With substantial progress in pharmaco- and psychotherapy of mood disorders, there is still an unmet need for patients regarding full remission and recovery.

According to STAR^*^D trial remission rates reach over 50% for the first two lines of treatment and reach two-thirds for four consecutive treatment steps (9). Yet even up to a third of patients do not remit and remain symptomatic despite the use of antidepressant therapy (ADT) (10–13). Such depression is generally referred to as treatment-resistant depression (TRD), albeit there is no consensus on the definition of this complex phenomenon (14). The regulatory definition of TRD is a failure of treatment with any two different antidepressants, given at an adequate dose and duration in the current depressive episode in patients with MDD (10, 13). However, less than a fifth of studies on TRD used all three commonly used criteria: a minimum of two prior treatment failures, an adequate drug dose, and a minimum duration of 4 weeks. Furthermore, 61% of studies did not mention an adequate dose and 70% did not describe the proper duration of treatment as inclusion criteria (14). The inconsistency in defining TRD and the heterogeneity of the study population poses a plethora of clinical and research challenges (15, 16). In recognition of these inconveniences, a new term “difficult-to-treat depression” was newly created (15–18). The elaboration on the concept is beyond the scope of the review.

Recent developments focus on TRD. Residual symptoms, however, are a separate field. The current definition for residual symptoms in MDD is formal remission with depressive symptoms at a subthreshold level (19). In past studies various definitions for residual symptoms were used: 17-item Hamilton Rating Scale for Depression (HAM-D-17) score between 8 and 18 (20), HAM-D-17 score equal to or below 10 (21), symptoms that persist after the response (improvement ≥ 50%) in validated scales (22, 23) or rating of at least 3 on the 7-points scale in the Clinical Interview for Depression (24). The broadest understanding of residual symptoms is the persistence of symptoms despite considerable clinical response to adequate therapy (25, 26). Furthermore, in older studies HAMD-D-17 score of 7 or less was tantamount to the absence of residual symptoms, thus patients were considered asymptomatic (20, 27).

The incidence of residual symptoms such as residual sleep disturbances, sad mood, loss of interest, loss of energy, weight changes or problems concentrating is high and can occur in over 90% of patients who reach formal remission (28). Since the 1990s it is known that residual symptoms contribute to a higher risk of relapse and recurrence, chronic course of illness, shorter duration between episodes, and worsen the long-term prognosis of MDD and should be directly targeted (4, 20, 29). It shall also be borne in mind that symptomatic and functional outcomes do not always overlap, with the former having higher response rates than the latter (30). Another noteworthy fact is that clinicians tend to rate patients' symptoms as less severe than they themselves do (31). Moreover, patients with residual symptoms report significant impairment of social and functional outcomes and are not satisfied with their quality of life (19, 32).

Insomnia is the subject of research as an independent phenomenon or co-occuring with other mental or somatic diseases. Insomnia complaints can be assessed by a handful of questionnaires. Pittsburgh Sleep Quality Index (PSQI) and Insomnia Severity Index (ISI) belong to the most widely used questionnaires with good psychometric properties to differentiate between good (i.e., PSQI < 5 or ISI < 8) and poor sleepers (33, 34).

Residual insomnia may be experienced in over 50% of the MDD population and is related to subsequent relapse and reduced quality of life (35, 36). In a re-analysis of STAR^*^D study early improvement in insomnia in a rater-based scale, unlike the self-report version, was associated with remission and response (37). Self-reported sleep efficiency might be influenced by a variety of psychosocial factors (e.g., low level of social support, more depressive symptoms, and overcommitment at work) (38). Despite the high prevalence of insomnia among residual symptoms and its clinical significance, this particular symptom seems to be relatively unaddressed in the literature.

Full remission and recovery of MDD should be the ultimate goal (32). We hypothesize the effective treatment of residual insomnia and a decrease in its incidence might reduce the risk of relapse and improve the functional outcome in MDD patients. Therefore, the main focus of this paper is to target residual insomnia and to identify effective methods in the treatment of residual insomnia in MDD patients with a secondary analysis of the most common residual insomnia subtypes.

## 2. Materials and methods

This systematic review was written according to the Preferred Reporting Items for Systematic Reviews and Meta-Analyses (PRISMA) statement (39). This review was not registered.

### 2.1. Information sources, search strategy, and selection process

On the 19th November 2022 electronic databases PubMed and Web of Science were searched for relevant papers. We used the following entries: “residual” and “insomnia” and “depression”, “residual” and “insomnia” and “major depressive disorder”, “residual” and “insomnia” and “major depression”, “residual” and “insomnia” and “mood disorder“, “residual” and “insomnia” and “bipolar depression”. The publication date was not limited.

Records were considered for inclusion if they met the following criteria:

1. Primary research article
2. AND
3. MDD diagnosis according to DSM (regardless of the edition) or ICD-10 criteria
4. AND
5. Treatment of residual insomnia
6. OR
7. Measurement of residual sleep disturbance.

**Figure 1:**
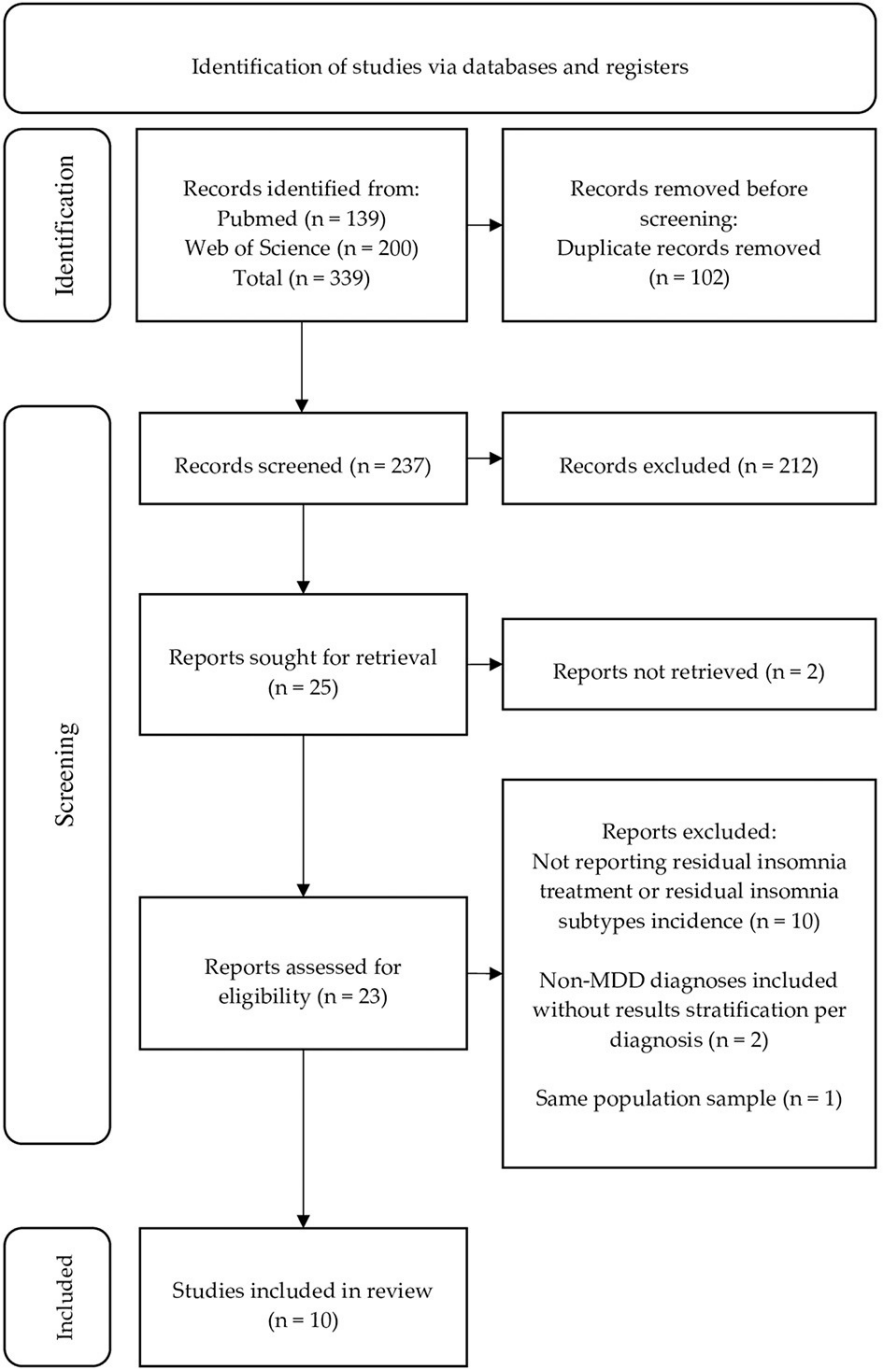
PRISMA flow chart representing the search strategy and the process of including studies for analysis.

For instance: (1 + 2 + 3) or (1 + 2 + 4). Details of the inclusion process are presented in the flow chart (Figure 1). Articles excluded did not report residual insomnia treatment nor the incidence of residual insomnia subtypes (10), included non-MDD patients without results stratification per diagnosis (2) and researched the same population (1). No studies involving patients with bipolar depression were found.

### 2.2. Data collection process

Both databases were searched by two independent reviewers: AK and AW Titles, abstracts, and full text were reviewed. Detected discrepancies were disputed between authors. In case of disagreement, the project co-supervisor (WC) was consulted until an agreement was reached.

The following data were extracted by both reviewers: authors, year of publication, study design, sample and control size, duration of the study, characteristic of the research and control group, type of the intervention (psychopharmacology, psychotherapy, other), outcomes (mean changes in sleep domain assessed per clinical scales or objective methods such as polysomnography).

### 2.3. Study risk of bias assessment

The risk of bias for randomized trials was evaluated using a method recommended by Cochrane System Reviewer Manual 5.1 (a revised tool to assess the risk of bias in randomized trials – RoB 2), including sequence generation, allocation concealment, blinding, missing outcome data, selective reporting, and other bias. The risk of bias may be assessed as “low”, “some concerns” or “high” (40, 41). To assess the risk of bias in non-randomized studies with intervention we used Newcastle-Ottawa Scale (42). This scale awards a maximum of nine points for each of the following items: selection (four stars), comparability (two stars), and outcomes (three stars). Studies with scores equal to or above seven points are considered “good quality” studies. At least two independent reviewers assessed the risk of bias for each study (JS and OS). Conflicting information was discussed to reach an agreement with the aid of the project co-supervisor (WC). The Robvis tool was used to illustrate the results from randomized trials (43).

### 2.4. Strategy for data synthesis

Due to the nature of the review and the heterogeneity of interventions included, data synthesis is mostly descriptive.

## 3. Results

### 3.1. Study selection

A total of 339 citations were identified in PubMed and Web of Science. The screening process is presented below in the form of a flow chart (Figure 1). Ten studies met the inclusion criteria. Four of them were randomized controlled trials, however, in one of them data on residual sleep disturbances were available only before randomization. One study had a within-subjects design and the remaining five were *post-hoc* analyses. They were grouped into two separate tables:

1. Studies on effective treatment methods for residual insomnia (Table 1).
2. Studies on the frequency of residual insomnia subtypes (Table 2).

**Table 1:** Studies on effective treatment methods for residual insomnia.

**References**	**Aim and study design**	**Population and number of subjects**	**Treatment groups and intervention**	**Sleep outcome measures**	**Outcome**
Mowla et al. (44)	RCT DB gabapentin vs. clonazepam for residual sleep disturbances	63 MDD with residual sleep disturbances	ADT + gabapentin 100–600 mg/d; ADT + clonazepam 0.5–2 mg/d	PSQI ISI	Sleep disturbances improved significantly in both groups; *p =* 0.001, effect size not stated No treatment effect in favor of the treatment strategy was observed (PSQI: *p =* 0.234; ISI: *p =* 0.456; effect sizes not stated)
Britton et al. (45)	RCT ADT + MBCT vs. standalone ADT in PRO and PSG	23 MDD with at least partial remission with ADT	ADT+MBCT; ADT alone	Sleep diary Polysomnography	Global improvement in favor of MBCT plus ADT in the following measures: Reduction in PSG total wake time *p =* 0.035, $\eta_{\text{p}}^{2}$ = 0.20 Sleep efficiency in PSG, *p =* 0.09, $\eta_{\text{p}}^{2}$ = 0.13 Total wake time measured by sleep diaries, *p =* 0.046, $\eta_{\text{p}}^{2}=$ 0.19 Sleep efficiency measured by sleep diaries, *p =* 0.007, $\eta_{\text{p}}^{2}$ = 0.14 Sleep diary: When TWT was decomposed into SOL and WASO, group X time interaction was close to significance for WASO (*p =* 0.053, $\eta_{\text{p}}^{2}$ = 0.18) but not for SOL (*p =* 0.24, $\eta_{\text{p}}^{2}=$ 0.07) PSG: When TWT was decomposed into SOL and WASO, group X time interactions were not significant (SOL: *p =* 0.24, $\eta_{\text{p}}^{2}$ = 0.067; WASO: *p =* 0.25, $\eta_{\text{p}}^{2}$ = 0.065).
Wing et al. (46)	RCT – blinded rater CBT for residual sleep disorders	66 (41 females) MDD with residual sleep disturbance	Usual treatment+ brief sleep focused CBT; usual treatment alone	PSQI ISI	No difference was observed between the groups. On *post-hoc* analysis results in favor of insomnia reduction for CBT group in 1 week FU and on 12-month FU.
Carney et al. (47)	CBT-D Prospective observational study, within-subjects design	24 (16 females) MDD	CBT-D, 20-week protocol based on “Mind over Mood”	PSQI	PSQI scores changed significantly from pre- to posttreatment, *p =* 0.002; $\eta_{\text{p}}^{2}$ =0.41 No difference was observed between pre- and posttreatment results (within analyzed parameters as stratified per PSQI score >5)
Rethorst et al. (48)	RCT for SSRI augmented with BA vs. SSRI standalone for PRO sleep quality measures in non-remitted MDD *Post-hoc* analysis	122 (100 females) non-remitted MDD	ADT + exercise with dose of 16 kilocalories per kilogram of body weight per week (KKW); ADT + with dose of 4 kilocalories per kilogram of body weight per week (KKW)	IDS-C30	Significant improvement in total insomnia (*p* < 0.0001; effect size not stated), mid-nocturnal and early-morning insomnia (p's < 0.035; effect size not stated), with no effect on sleep onset insomnia (*p* = 0.12; effect size not stated) No significant differences between exercise treatment groups on total insomnia score or on any individual sleep item.

ADT, antidepressant therapy; BA, behavioral activation; CBT, cognitive behavioral therapy; CBT-D, cognitive behavioral therapy for depression; DB, double blind; FU, follow up; IDS-C30, Inventory for Depression Symptomatology-Clinician 30-item; ISI, Insomnia Severity Index; KKW, kilocalories per kilogram of body weight per week; MBCT, mindfulness-based cognitive therapy; MDD, major depressive disorder; PRO, patient reported outcome; PSQI, Pittsburgh Sleep Quality Index; RCT, randomized controlled trial; SOL, sleep onset latency; SSRI, selective serotonin reuptake inhibitor; TWT, total wake time; WASO, wake after sleep onset.

**Table 2:** Studies on the frequency of residual insomnia subtypes.

**References**	**Aim and Study design**	**Population and number of subjects**	**Treatment groups and intervention**	**Sleep outcome measures**	**Outcome**
Taylor et al. (49)	RCT CT for residual symptoms in MDD Sleep data extracted for study endpoint for aCT	84 (61 female) with recurrent MDD who responded to 20 sessions of aCT	aCT	HAM-D	Patients after treatment with aCT reported following difficulties: early insomnia (17%), middle insomnia (36%), late insomnia (24%).
McClintock et al. (22)	Exploratory study for residual symptoms persistence in MDD ADT *Post-hoc* analysis, within subjects	428 (284 female) MDD who have responded but not remitted.	Citalopram 20–60 mg/d up to 14 weeks	QIDS-SR	Improvement in: sleep onset insomnia (82.9%) at baseline -> sleep onset insomnia persistent at exit (57.5%) Mid-nocturnal insomnia at baseline (91.4%) -> mid-nocturnal insomnia persistent at exit (81.6%) Early morning insomnia at baseline (69.6%) -> early morning insomnia persistent at exit (49.0%)
Nierenberg et al. (28)	Exploratory study for residual symptoms persistence in MDD ADT *Post-hoc* analysis, within subjects	943 MDD with residual symptoms, currently in remission	Citalopram 20–60 mg/d for up to 14 weeks	QIDS-SR	The most frequent and persistent sleep residual symptoms were mid-nocturnal insomnia (58.8%), sleep onset insomnia (35.8%), early morning insomnia (21.1%).
Sakurai et al. (35)	Exploratory study for residual symptoms persistence in MDD ADT *Post-hoc* analysis, within subjects	1133 (747 female) MDD after acute treatment, who entered follow-up phase, currently in remission	Citalopram 20–60 mg/d for up to 14 weeks	QIDS-SR QIDS-C	QIDS-SR: sleep onset insomnia 34.5% mid-nocturnal insomnia 74.1% early morning insomnia 18.2% QIDS-C: sleep onset insomnia 29.6% mid-nocturnal insomnia 58.2% early morning insomnia 18.2% Differences in sleep onset insomnia and mid-nocturnal insomnia in QIDS-SR and QIDS-C were statistically relevant (*p =* 0.001 and *p < * 0.001 respectively)
Iovieno et al. (50)	RCT DB treatment with fluoxetine and subsequent relapse *Post-hoc* analysis, within subjects	203 (117 female) MDD with residual symptoms, currently in remission	Fluoxetine up to 60 mg/d for 12 weeks	HAM-D for response and remission HAM-D-28 for residual symptoms	Fluoxetine remitters reporting symptoms of insomnia across treatment timeline (baseline to endpoint at week 12) early insomnia (57.1% -> 24.1%), middle insomnia (63.1% -> 37.5%), late insomnia (52.7% -> 29.9%).

aCT, acute cognitive therapy; ADT, antidepressant therapy; CT, cognitive therapy; HAM-D, Hamilton Depression Rating Scale 17-item; HAM-D-28, Hamilton Depression Rating Scale 28-item; MDD, major depressive disorder; RCT, randomized controlled trial; QIDS-C, Quick Inventory for Depression Symptomatology Clinician; QIDS-SR, Quick Inventory for Depression Symptomatology Self-Report.

### 3.2. Risk of bias in the studies

**Figure 2:**
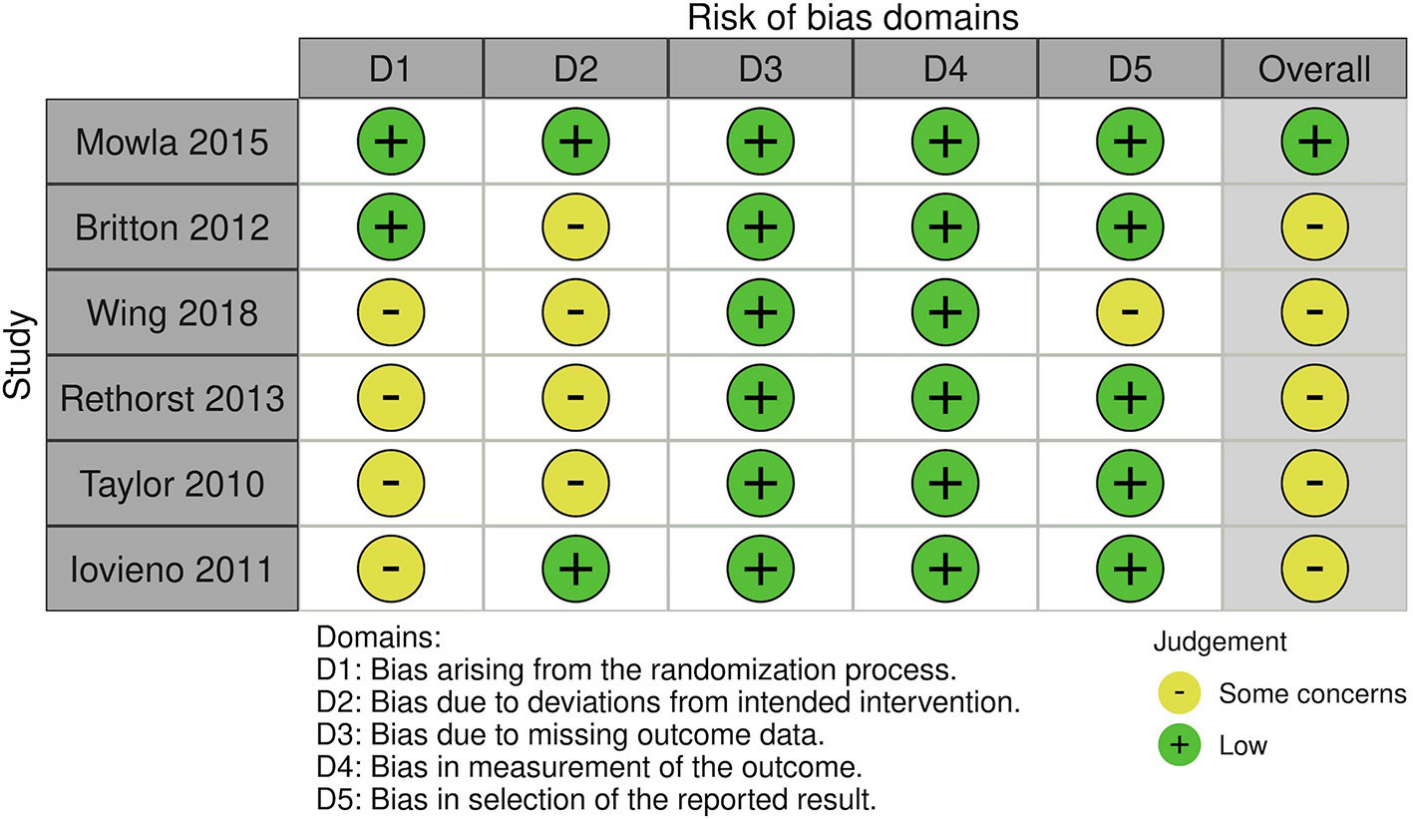
Risk of bias in randomized controlled trials using RoB 2 tool.

**Figure 3:**
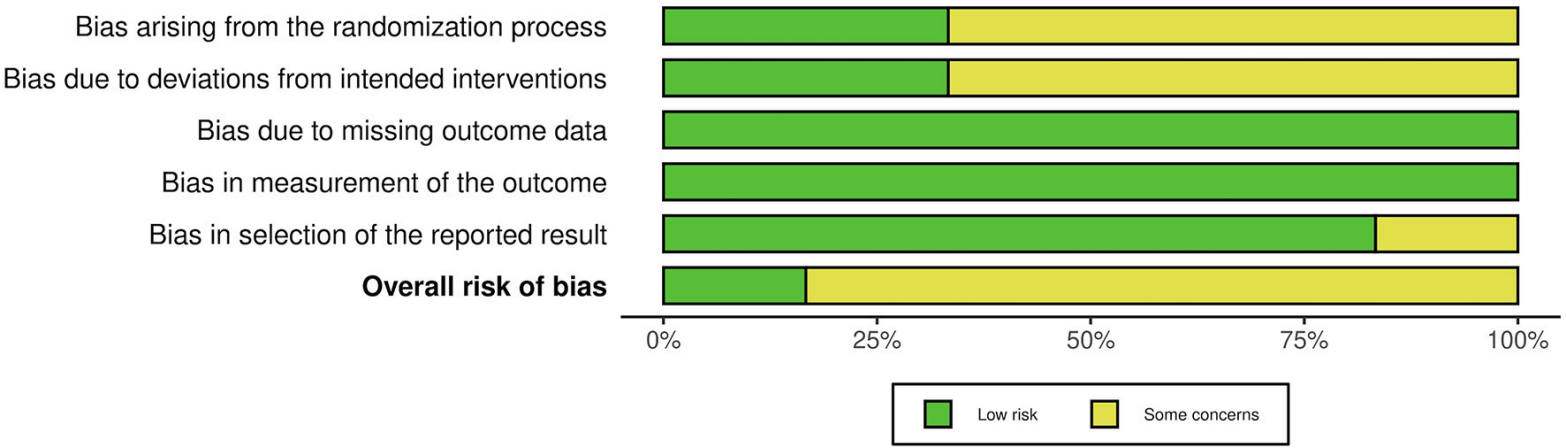
Risk of bias graph showing authors' assessment of risk of bias across all included studies.

**Figure 4:**
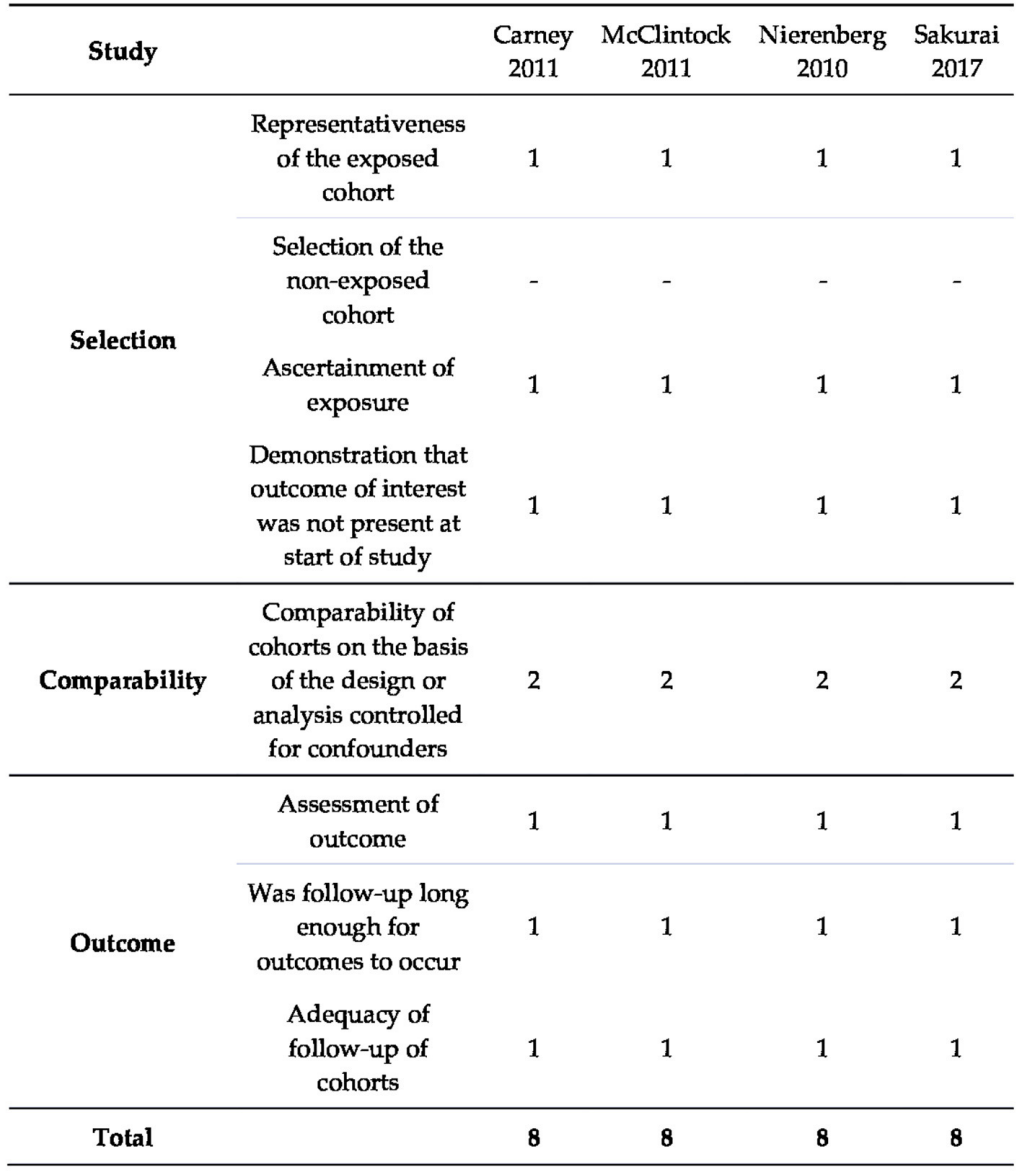
Risk of bias in non-randomized studies evaluated using Newcastle–Ottawa Scale.

The risk of bias assessment in the studies using the RoB 2 tool and Newcastle-Ottawa Scale is presented in Figures 2–4.

### 3.3. Study characteristics

Studies included in the systematic review are presented below in Tables 1, 2.

### 3.4. Studies on residual insomnia treatment

#### 3.4.1. Psychopharmacology

In a randomized, controlled, double-blind study (44), 63 MDD patients with residual sleep disturbances (PSQI >5 or ISI > 8) were randomized to receive either gabapentin in doses (100–600 mg/d) or clonazepam (0.5–2 mg/d). After 4 weeks both groups improved in both aforementioned scales, without showing any statistical difference between each drug, which proves gabapentin may be equally efficacious as clonazepam in treating residual sleep disturbances.

#### 3.4.2. Psychotherapy

In another study (45), 23 MDD patients with at least partial remission were randomized to receive 8-week mindfulness meditation training together with ADT in order to observe its influence on self-reported and objectively measured sleep profiles. It turned out that mindfulness-based cognitive therapy (MBCT) reduced total wake time and improved sleep efficiency both in the sleep diary and in polysomnography. However, when total wake time was decomposed into sleep onset latency (sleep initiation) and wake after sleep onset (sleep maintenance), the result was of no statistical significance. Furthermore, MBCT did not have any impact on sleep depth or architecture.

In a randomized controlled, assessor-blind, parallel-group study conducted by Wing et al. (46), 66 patients were randomized to a group with sleep-focused cognitive behavioral therapy (CBT) plus ADT or to usual treatment alone. The first group had a higher reduction in ISI at the 1-week follow-up. Nevertheless, the difference between the two groups did not reach significance at subsequent follow-ups. In the author's point of view, results do not stem from the lack of efficacy of the intervention, but rather from the improvement in the control group. They argue it could be due to the prescription of hypnotics from physicians in outpatient clinics. In comparison, the intervention group had a better outcome in PSQI at any time from 1-week to 12-month follow-up.

In a study by Carney et al. (47), 24 patients with MDD diagnosis were treated with cognitive behavioral therapy for depression (CBT-D) in a 20-week protocol “Mind over Mood”. Sleep improvement measured by PSQI was of statistical significance, nonetheless, 91.3% of the subjects still had PSQI score >5, whereas at baseline it was 96.4%. Despite statistical significance, authors suggest CBT-D might not be sufficient at alleviating residual insomnia, given the high rate of reported posttreatment symptoms.

#### 3.4.3. Other

The effect of exercise with concomitant ADT on self-reported sleep quality was the subject of a *post-hoc* analysis conducted by Rethorst et al. (48). One hundred twenty-two patients were randomized to 4 or 16 kilocalories per kilogram of body weight per week for 12 weeks of aerobic exercise as treatment augmentation. Exercise sessions were supervised a minimum of once per week throughout the entire trial. The outcome was tested using the Inventory of Depressive Symptomatology–Clinician (IDS-C30) sleep items. Significant reduction in symptom severity concerned total insomnia, mid-nocturnal and early-morning insomnia. Exercise treatment groups did not differ statistically.

### 3.5. Incidence of residual insomnia subtypes

Another randomized controlled trial consisted of 84 patients with recurrent MDD who responded to 12 to 14-week acute cognitive therapy (aCT) protocol and were subsequently randomized to either continuation phase therapy or evaluation only (49). Sleep data on residual insomnia were available only after aCT and before randomization. In HAM-D sleep items (early, middle, late insomnia) responders reported frequency of 17, 36, and 24% respectively.

The STAR^*^D study group provided two *post-hoc* analyses of this clinical trial focused on residual sleep disturbances in different subgroups of subjects after initial treatment with citalopram. The first analysis (22) concerned 428 participants, who responded, but did not remit. Out of patients who reported sleep complaints at baseline, nearly half (49%) continued to suffer from early-morning insomnia and more than half (57.5%) from sleep onset insomnia. Mid-nocturnal insomnia persisted in over 81.6% of participants. In those, who did not have sleep complaints at baseline, the following sleep disturbances emerged during treatment: mid-nocturnal insomnia (51.4%), sleep onset insomnia (26.0%), and early-morning insomnia (13.9%). The second analysis (28) investigated the incidence rate of residual symptoms in remitters. Similarly, patients most frequently had persistent mid-nocturnal insomnia (58.8%), followed by sleep onset insomnia (35.8%) and early morning insomnia (21.1%). The total prevalence of residual insomnia was higher due to the emergence of sleep onset insomnia (9.0%), mid-nocturnal insomnia (23.8%), and early morning insomnia (10.0%) in patients, whose sleep was not impaired at baseline. The authors underline the importance of the distinction between “true” residual and treatment-emergent symptoms, which may be considered adverse events of ADT.

Yet another re-analysis of the STAR^*^D study by Japanese researchers (35) tried to determine which residual symptoms, assessed by self-report and clinician rating, contribute to the subsequent relapse. Self-assessed and rater-based data of 1,133 patients who entered the naturalistic follow-up phase were available. Again, mid-nocturnal residual insomnia was the most prevalent subtype of residual sleep disturbances. It should be emphasized that clinicians rated sleep complaints less severely than patients themselves did.

The last study included in the review is a *post-hoc* analysis of a study that investigated if the initial effect of acute treatment of MDD could be a reliable predictor of the long-term outcome (50). Participants were treated with fluoxetine for 12 weeks. Those who responded qualified for the discontinuation phase and were randomized into two groups. Data on residual sleep disturbance are available for remitters only and at the time of randomization. Symptoms were measured on the Hamilton Depression Rating Scale 28-item scale (HAM-D-28). Again, the most prevalent and persistent was middle insomnia (37.5%), followed by late insomnia (29.9%) and early insomnia (24.1%). Similarly to studies included, there were participants who developed insomnia during treatment without having it at baseline, i.e., middle insomnia (26.7%), early (12.6%), and late insomnia (14.6%).

## 4. Discussion

Residual insomnia in MDD may effectively be treated with non-pharmacological interventions, with some evidence in favor of pharmacological treatment being efficacious (Table 3). There is robust evidence that CBT reduces both depression and insomnia severity and provides better durability of improvement than medication (51–53).

**Table 3:** Possible residual insomnia treatment.

**Intervention**	**Example**
Psychopharmacology	Gabapentin Clonazepam (short-term only)
Psychotherapy	CBT MBCT
Other	BA

CBT, cognitive behavioral therapy; MBCT, mindfulness-based cognitive therapy; BA, behavioral activation.

Nevertheless, the outcome may be different for CBT-D and cognitive behavioral therapy for insomnia (CBT-I). The first significantly reduces depressive symptoms, but the improvement in insomnia symptoms is not satisfactory (47, 54). On the other hand, CBT-I significantly alleviates residual insomnia and the onset of action is noticeable within a week (46, 51, 53, 54). It takes only between 6 and 8 sessions and may provide sustainable remission of symptoms (46, 51, 52, 54, 55).

Mindfulness has been proven to increase self-reported and objectively-measured sleep efficiency and to reduce total wake time in MDD patients with residual insomnia. However, it was used as an augmentation of ADT (45). This is consistent with the literature, that mindfulness meditation might be considered as an augmentation therapy (56). There is evidence that it might be ineffective compared to evidence-based sleep treatments but might be superior to non-specific controls such as waitlist or attention/education controls (57, 58). Behavioral activation (BA) as an adjuvant depression therapy is supported by some studies (59, 60). One *post-hoc* analysis included in this review produced promising results in mitigating residual insomnia and the improvement did not depend on the BA intensity (48). Future studies should focus on clarifying the relationship between exercise, depression, and insomnia. Only one study targeted residual sleep disturbance with pharmacological treatment and used gabapentin. This treatment proved to be equally efficacious as clonazepam. In contrast to benzodiazepines, the potential for addiction is low (61, 62). We have not found any data regarding residual insomnia treatment with the medication commonly used to treat insomnia. Since residual symptoms are not easily treated, it may not be taken for granted, that they are equally efficacious in residual insomnia treatment.

All studies that measured the incidence of sleep disturbances showed mid-nocturnal insomnia is the most prevalent residual insomnia subtype (36–81.6%), followed by sleep onset insomnia (17.0–57.5%) and early-morning insomnia (18.2–49.0%), depending on the study (Table 4) (22, 28, 35, 49, 50). Higher rates of residual insomnia seem to appear in responders and are less prevalent in remitters (25). Treatment-emergent symptoms are a separate phenomenon and should be distinguished from persistent residual symptoms. The differentiation seems essential, as “true” residual insomnia rates may be exaggerated and a proportion of them should be classified as adverse effects of ADT rather than the consequence of the disease. Although sedating antidepressants such as trazodone, amitriptyline or doxepin promote sleep and may be used off-label as hypnotics in MDD, it is proven that some antidepressants disrupt sleep and may induce primary sleep disorders (e.g., restless leg syndrome) (63). Better categorization might bring more attention to the origin of symptoms and enable optimization of the treatment. In general, this may apply to all residual symptoms.

**Table 4:** Incidence of residual insomnia subtypes.

**Residual insomnia subtype**	**Incidence**
Sleep onset insomnia	17.0–57.5%
Mid-nocturnal insomnia	36.0–81.6%
Early-morning insomnia	18.2–49.0%

A possible confounding factor that we can indicate is that in a study by Mowla et al. (44), the ADT addition benzodiazepine was clonazepam, which is not recommended for insomnia, although several studies have confirmed the efficacy of clonazepam with typical doses (64). Another one is the assessment of sleep quality using only IDS-C30 (48). Although it consists of four sleep items, it was originally created to rate depressive symptoms and might miss out on the full scope of sleep symptomatology (65, 66). Overall, there are few studies that specifically target residual insomnia as a primary outcome. The ones that do exist were carried out on relatively small populations, which may rather propose future direction of research than decisively conclude what methods are effective. Finally, few studies measure residual insomnia subtypes. What is more, not all of them distinguish between persistent and emergent symptoms. Almost all sleep data on residual insomnia subtypes rely on self-report and future objectivization might increase knowledge of residual sleep disturbances. Still, it should be kept in mind the diagnosis of insomnia is based on patient's dissatisfaction with sleep quality or quantity and is strictly subjective, there are no objective criteria. The above-mentioned limitations shall be indicative of future trial designs. Furthermore, the variability of residual symptom definitions in MDD may produce an inhomogeneity of the results obtained. Thus, the conclusions drawn from comparative trials may produce ambiguity and shall not be generalized. It seems warranted, the regulatory and/or professional definition of residual MDD symptoms might contribute to the quality of future research. Besides, residual symptoms in MDD shall not be confused with TRD which comprises different entities with the regulatory definition (10). Briefly, residual symptoms are symptoms that persist after achieving formal remission criteria, whereas TRD is the persistence of symptoms without reaching remission, despite adequate dose and duration of ADT according to the regulatory definition (10, 13, 19). Thus, TRD and residual symptoms are two distinct phenomena, that shall not be confused and require clinical differentiation per definition and anticipated treatment outcome.

## 5. Conclusions

As residual insomnia in MDD is a common and burdensome phenomenon impacting poor prognosis, limited data support evidence for the use of CBT, MBCT, and BA. Some evidence supports the use of GABAergic medication, however a serious limitation is a short time-frame of the intervention. Regulatory and/or clinical definition of residual symptoms in MDD is needed. More clinical and experimental data regarding residual insomnia treatment may contribute to the recovery and long-Term wellbeing of MDD patients.

## Data availability statement

The original contributions presented in the study are included in the article/Supplementary material, further inquiries can be directed to the corresponding author.

## Author contributions

Conceptualization: WC and AK. Methodology and writing–original draft preparation: AK. Software: AW. Validation: WC, AW, and AK. Formal analysis and investigation: AK, AW, JS, and OS. Writing–review and editing: WC, AW, and AD. Visualization: AD. Supervision and funding acquisition: WC. All authors have read and agreed to the published version of the manuscript.
